# 5-(4-Fluoro­phen­yl)-5-methyl­imidazolidine-2,4-dione

**DOI:** 10.1107/S1600536807067803

**Published:** 2008-01-16

**Authors:** M. Kaleem Kashif, Abid Hussain, M. Khawar Rauf, Masahiro Ebihara, Shahid Hameed

**Affiliations:** a Department of Chemistry, Quaid-i-Azam University, Islamabad 45320, Pakistan; b Department of Chemistry, Faculty of Engineering, Gifu University, Yanagido, Gifu 501-1193, Japan

## Abstract

In the title compound, C_10_H_9_FN_2_O_2_, the dihedral angle between the hydantoin unit and the benzene ring is 65.55 (5)°. The atoms in the hydantoin ring are coplanar, with a mean deviation of 0.015 Å and a maximum deviation of 0.075 (2) Å for one carbonyl O atom. N—H⋯O hydrogen bonds link the mol­ecules into one-dimensional chains, with one carbonyl group acting as a bifurcated acceptor and the other accepting no hydrogen bonds.

## Related literature

For related literature, see: Ahmad *et al.* (2000 ▶, 2002 ▶); Balavoine *et al.* (2007 ▶); Mullica *et al.* (1998 ▶); Park *et al.* (2007 ▶); Rajic *et al.* (2006 ▶); Sheppeck *et al.* (2007 ▶).
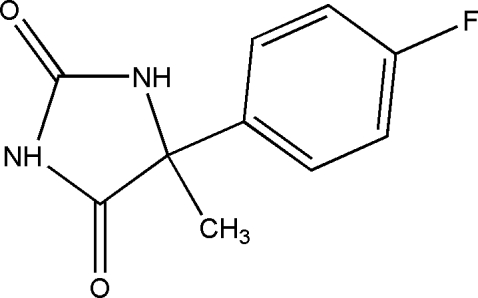


## Experimental

### 

#### Crystal data

C_10_H_9_FN_2_O_2_
*M* *_r_* = 208.19
Orthorhombic, 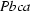

*a* = 7.096 (2) Å
*b* = 11.348 (3) Å
*c* = 22.661 (7) Å
*V* = 1824.7 (10) Å^3^
*Z* = 8
Mo *K*α radiation
μ = 0.12 mm^−1^
*T* = 123 (2) K
0.34 × 0.30 × 0.20 mm

#### Data collection

Rigaku Mercury CCD diffractometer
Absorption correction: none
13516 measured reflections
2083 independent reflections
2054 reflections with *I* > 2σ(*I*)
*R*_int_ = 0.026

#### Refinement

*R*[*F* ^2^ > 2σ(*F* ^2^)] = 0.045
*wR*(*F* ^2^) = 0.094
*S* = 1.22
2083 reflections
145 parameters
H atoms treated by a mixture of independent and constrained refinement
Δρ_max_ = 0.31 e Å^−3^
Δρ_min_ = −0.16 e Å^−3^

### 

Data collection: *CrystalClear* (Molecular Structure Corporation & Rigaku, 2001 ▶); cell refinement: *CrystalClear*; data reduction: *TEXSAN* (Rigaku/MSC, 2004 ▶); program(s) used to solve structure: *SIR97* (Altomare *et al.*, 1999 ▶); program(s) used to refine structure: *SHELXL97* (Sheldrick, 1997 ▶); molecular graphics: *ORTEPII* (Johnson, 1976 ▶); software used to prepare material for publication: *SHELXL97* and *TEXSAN*.

## Supplementary Material

Crystal structure: contains datablocks I, global. DOI: 10.1107/S1600536807067803/bi2269sup1.cif

Structure factors: contains datablocks I. DOI: 10.1107/S1600536807067803/bi2269Isup2.hkl

Additional supplementary materials:  crystallographic information; 3D view; checkCIF report

## Figures and Tables

**Table 1:** Hydrogen-bond geometry (Å, °)

*D*—H⋯*A*	*D*—H	H⋯*A*	*D*⋯*A*	*D*—H⋯*A*
N1—H1⋯O1^i^	0.88 (2)	2.04 (2)	2.8834 (17)	160.5 (18)
N2—H2⋯O1^ii^	0.89 (2)	1.96 (2)	2.8318 (17)	165.9 (17)

Symmetry codes: (i) 
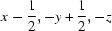
; (ii) 
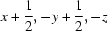
.

**Fig. 1.**
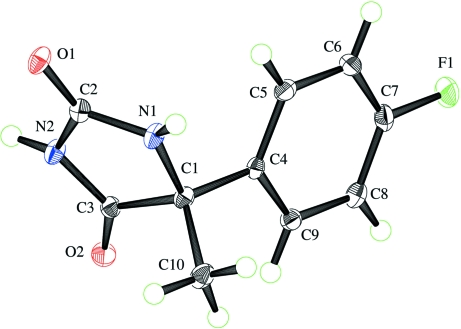
Molecular structure showing displacement ellipsoids at the 30% probability level for non-H atoms.

## supplementary crystallographic information

### Comment

Active research is being carried out in this laboratory on the synthesis of sulfonyl cyclic ureas and their evaluation as hypoglycemic agents (Ahmad *et al.*, 2002; Ahmad *et al.*, 2000). Imidazolidine-2,4-diones, a class of cyclic urea molecules, exhibit diverse biological activities like anti-cancer (Sheppeck *et al.*, 2007), anti-viral (Rajic *et al.*, 2006), COX-2 inhibitors (Park *et al.*, 2007) and hormone receptor antagonists (Balavoine *et al.*, 2007). The title compound (Fig. 1) was synthesized as an intermediate for onward conversion to sulfonyl derivatives for hypoglycemic assay. It contains a hydantoin ring attached to a methyl and *p*-flourophenyl group at the chiral centre C1. All bond distances are in agreement with experimental values found in similar compounds. The atoms in the hydantoin ring are planar as expected (Mullica *et al.*, 1998) with a mean standard deviation of 0.015 Å. The C2—O1 and C3—O2 bond distances are 1.2320 (17) Å and 1.2080 (17) Å, respectively, which are close to the standard value for C?O (1.20 Å). The dihedral angle subtended by the *p*-flourophenyl group at the chiral centre C(1) is 65.55 (5)°.

### Experimental

4-Fluoroacetophenone (0.1 mol) and ammonium carbonate (0.6 mol) were placed in a 100 ml round bottom flask. Potassium cyanide (0.1 mol) was dissolved in aqueous ethanol (60%) and added to the reaction flask. The mixture was heated on an oil bath at 328–333 K until the reaction was completed (monitored by TLC). After cooling to room temperature, the reaction mixture was concentrated and acidified using conc. HCl. The resulting precipitate was filtered, dissolved in saturated NaOH(aq) solution and extracted with diethyl ether (25 ml). The aqueous layer was acidified to precipitate the title compound, which was filtered, dried and recrystallized from ethanol/water. Yield: 75%; m.p. 485–488 K; *R*_f_ (pet. ether/ethyl acetate 1:2) 0.58.

IR (KBr, ν_max_, cm^-1^): 3412, 3245, 3058, 2989, 1773, 1719, 1602, 1378, 1274, 838; ^1^H-NMR (acetone-*d6*) δ: 1.80 (3*H*, s), 7.18 (2*H*, m), 7.64 (2*H*, m), 7.71 (1*H*, bs), 9.72 (1*H*, bs); EIMS (m/*z*, %): 208 (*M*^+^, 20), 193 (65), 165 (5), 137 (36), 122 (100), 95 (25); Elemental analysis calculated: C 57.69, H 4.36, N 13.46; found: C 57.62, H 4.38, N 13.60%.

### Refinement

H atoms bound to N atoms were located in difference Fourier maps and refined freely with isotropic displacement parameters. Other H atoms were placed in idealized positions and treated as riding, with C—H = 0.95–0.98 Å and with *U*_iso_(H) = 1.2 or 1.5*U*_eq_(C).

### Crystal data

**Table:**

C_10_H_9_FN_2_O_2_	*F*_000_ = 864
*M**_r_* = 208.19	*D*_x_ = 1.516 Mg m^−^^3^
Orthorhombic, *P**b**c**a*	Mo *K*α radiation λ = 0.71070 Å
Hall symbol: -P 2ac 2ab	Cell parameters from 5426 reflections
*a* = 7.096 (2) Å	θ = 3.0–27.5º
*b* = 11.348 (3) Å	µ = 0.12 mm^−^^1^
*c* = 22.661 (7) Å	*T* = 123 (2) K
*V* = 1824.7 (10) Å^3^	Block, colorless
*Z* = 8	0.34 × 0.30 × 0.20 mm

### Data collection

**Table:**

Rigaku Mercury CCD diffractometer	2054 reflections with *I* > 2σ(*I*)
Monochromator: graphite	*R*_int_ = 0.026
Detector resolution: 14.62 pixels mm^-1^	θ_max_ = 27.5º
*T* = 123(2) K	θ_min_ = 3.5º
ω scans	*h* = −9→8
Absorption correction: none	*k* = −14→13
13516 measured reflections	*l* = −17→29
2083 independent reflections	

### Refinement

**Table:**

Refinement on *F*^2^	Secondary atom site location: difference Fourier map
Least-squares matrix: full	Hydrogen site location: inferred from neighbouring sites
*R*[*F*^2^ > 2σ(*F*^2^)] = 0.045	H atoms treated by a mixture of independent and constrained refinement
*wR*(*F*^2^) = 0.094	*w* = 1/[σ^2^(*F*_o_^2^) + (0.028*P*)^2^ + 1.2798*P*] where *P* = (*F*_o_^2^ + 2*F*_c_^2^)/3
*S* = 1.22	(Δ/σ)_max_ = 0.001
2083 reflections	Δρ_max_ = 0.31 e Å^−^^3^
145 parameters	Δρ_min_ = −0.16 e Å^−^^3^
Primary atom site location: structure-invariant direct methods	Extinction correction: none

### Special details

**Table:**

Geometry. All e.s.d.'s (except the e.s.d. in the dihedral angle between two l.s. planes) are estimated using the full covariance matrix. The cell e.s.d.'s are taken into account individually in the estimation of e.s.d.'s in distances, angles and torsion angles; correlations between e.s.d.'s in cell parameters are only used when they are defined by crystal symmetry. An approximate (isotropic) treatment of cell e.s.d.'s is used for estimating e.s.d.'s involving l.s. planes.
Refinement. Refinement of *F*^2^ against ALL reflections. The weighted *R*-factor *wR* and goodness of fit *S* are based on *F*^2^, conventional *R*-factors *R* are based on *F*, with *F* set to zero for negative *F*^2^. The threshold expression of *F*^2^ > σ(*F*^2^) is used only for calculating *R*-factors(gt) *etc*. and is not relevant to the choice of reflections for refinement. *R*-factors based on *F*^2^ are statistically about twice as large as those based on *F*, and *R*- factors based on ALL data will be even larger.

### Fractional atomic coordinates and isotropic or equivalent isotropic displacement parameters (Å^2^)

**Table:**

	*x*	*y*	*z*	*U*_iso_*/*U*_eq_	
C1	0.13701 (19)	0.06340 (11)	0.10194 (6)	0.0127 (3)	
N1	0.09007 (16)	0.16162 (10)	0.06286 (5)	0.0141 (2)	
H1	−0.025 (3)	0.1859 (17)	0.0547 (9)	0.028 (5)*	
C2	0.23589 (19)	0.19597 (12)	0.02932 (6)	0.0135 (3)	
O1	0.23772 (14)	0.27474 (9)	−0.00808 (4)	0.0167 (2)	
N2	0.38961 (17)	0.12558 (10)	0.04332 (5)	0.0151 (3)	
H2	0.504 (3)	0.1445 (16)	0.0305 (8)	0.024 (5)*	
C3	0.34432 (19)	0.04077 (11)	0.08415 (6)	0.0129 (3)	
O2	0.44614 (15)	−0.03631 (8)	0.10246 (4)	0.0177 (2)	
C4	0.13117 (18)	0.09904 (11)	0.16706 (6)	0.0118 (3)	
C5	0.08781 (19)	0.21387 (12)	0.18428 (6)	0.0150 (3)	
H5	0.0607	0.2719	0.1553	0.018*	
C6	0.0841 (2)	0.24367 (12)	0.24395 (6)	0.0175 (3)	
H6	0.0542	0.3217	0.2560	0.021*	
C7	0.1246 (2)	0.15835 (13)	0.28513 (6)	0.0170 (3)	
C8	0.1685 (2)	0.04404 (12)	0.26982 (6)	0.0158 (3)	
H8	0.1954	−0.0134	0.2992	0.019*	
C9	0.17244 (19)	0.01526 (12)	0.21023 (6)	0.0139 (3)	
H9	0.2037	−0.0628	0.1987	0.017*	
C10	0.0141 (2)	−0.04446 (12)	0.08893 (6)	0.0183 (3)	
H10A	−0.1179	−0.0258	0.0975	0.027*	
H10B	0.0549	−0.1105	0.1137	0.027*	
H10C	0.0267	−0.0660	0.0472	0.027*	
F1	0.12297 (14)	0.18871 (8)	0.34320 (4)	0.0258 (2)	

### Atomic displacement parameters (Å^2^)

**Table:**

	*U*^11^	*U*^22^	*U*^33^	*U*^12^	*U*^13^	*U*^23^
C1	0.0126 (6)	0.0140 (6)	0.0116 (6)	−0.0001 (5)	−0.0008 (5)	0.0028 (5)
N1	0.0104 (5)	0.0188 (6)	0.0132 (5)	0.0008 (4)	−0.0007 (4)	0.0057 (4)
C2	0.0120 (6)	0.0168 (6)	0.0115 (6)	−0.0011 (5)	−0.0016 (5)	−0.0003 (5)
O1	0.0127 (5)	0.0210 (5)	0.0166 (5)	0.0002 (4)	0.0000 (4)	0.0079 (4)
N2	0.0112 (6)	0.0183 (6)	0.0159 (6)	0.0004 (4)	0.0011 (4)	0.0053 (5)
C3	0.0145 (6)	0.0138 (6)	0.0105 (6)	−0.0007 (5)	0.0001 (5)	−0.0003 (5)
O2	0.0183 (5)	0.0163 (5)	0.0186 (5)	0.0042 (4)	0.0014 (4)	0.0030 (4)
C4	0.0092 (6)	0.0147 (6)	0.0115 (6)	−0.0016 (5)	0.0008 (5)	0.0009 (5)
C5	0.0145 (6)	0.0137 (6)	0.0169 (6)	−0.0010 (5)	0.0012 (5)	0.0026 (5)
C6	0.0172 (7)	0.0145 (6)	0.0209 (7)	−0.0014 (5)	0.0052 (5)	−0.0037 (5)
C7	0.0155 (7)	0.0229 (7)	0.0125 (6)	−0.0043 (5)	0.0030 (5)	−0.0042 (5)
C8	0.0147 (6)	0.0194 (6)	0.0132 (6)	−0.0015 (5)	0.0001 (5)	0.0037 (5)
C9	0.0136 (6)	0.0133 (6)	0.0148 (6)	0.0002 (5)	0.0007 (5)	0.0009 (5)
C10	0.0200 (7)	0.0196 (7)	0.0152 (6)	−0.0070 (5)	−0.0006 (5)	−0.0002 (5)
F1	0.0347 (5)	0.0288 (5)	0.0140 (4)	−0.0055 (4)	0.0051 (4)	−0.0061 (4)

### Geometric parameters (Å, °)

**Table:**

C1—N1	1.4620 (17)	C5—C6	1.394 (2)
C1—C4	1.5306 (18)	C5—H5	0.950
C1—C10	1.5316 (19)	C6—C7	1.375 (2)
C1—C3	1.5468 (19)	C6—H6	0.950
N1—C2	1.3417 (18)	C7—F1	1.3604 (16)
N1—H1	0.88 (2)	C7—C8	1.378 (2)
C2—O1	1.2320 (17)	C8—C9	1.3897 (19)
C2—N2	1.3887 (18)	C8—H8	0.950
N2—C3	1.3732 (17)	C9—H9	0.950
N2—H2	0.89 (2)	C10—H10A	0.980
C3—O2	1.2080 (17)	C10—H10B	0.980
C4—C5	1.3946 (19)	C10—H10C	0.980
C4—C9	1.3952 (18)		
			
N1—C1—C4	112.11 (11)	C6—C5—C4	120.14 (13)
N1—C1—C10	111.28 (11)	C6—C5—H5	119.9
C4—C1—C10	112.42 (11)	C4—C5—H5	119.9
N1—C1—C3	100.68 (10)	C7—C6—C5	118.92 (13)
C4—C1—C3	108.71 (10)	C7—C6—H6	120.5
C10—C1—C3	111.03 (11)	C5—C6—H6	120.5
C2—N1—C1	112.89 (11)	F1—C7—C6	118.45 (13)
C2—N1—H1	120.3 (13)	F1—C7—C8	118.92 (13)
C1—N1—H1	125.2 (13)	C6—C7—C8	122.62 (13)
O1—C2—N1	127.49 (13)	C7—C8—C9	118.05 (12)
O1—C2—N2	124.48 (12)	C7—C8—H8	121.0
N1—C2—N2	108.02 (11)	C9—C8—H8	121.0
C3—N2—C2	111.91 (11)	C8—C9—C4	121.13 (12)
C3—N2—H2	127.1 (12)	C8—C9—H9	119.4
C2—N2—H2	120.3 (12)	C4—C9—H9	119.4
O2—C3—N2	126.79 (13)	C1—C10—H10A	109.5
O2—C3—C1	126.84 (12)	C1—C10—H10B	109.5
N2—C3—C1	106.37 (11)	H10A—C10—H10B	109.5
C5—C4—C9	119.13 (12)	C1—C10—H10C	109.5
C5—C4—C1	121.51 (12)	H10A—C10—H10C	109.5
C9—C4—C1	119.35 (12)	H10B—C10—H10C	109.5
			
C4—C1—N1—C2	113.75 (13)	C10—C1—C4—C5	−127.12 (14)
C10—C1—N1—C2	−119.38 (13)	C3—C1—C4—C5	109.54 (14)
C3—C1—N1—C2	−1.65 (14)	N1—C1—C4—C9	179.95 (12)
C1—N1—C2—O1	178.84 (13)	C10—C1—C4—C9	53.70 (17)
C1—N1—C2—N2	−0.49 (15)	C3—C1—C4—C9	−69.63 (15)
O1—C2—N2—C3	−176.54 (13)	C9—C4—C5—C6	−0.6 (2)
N1—C2—N2—C3	2.81 (16)	C1—C4—C5—C6	−179.82 (12)
C2—N2—C3—O2	176.10 (13)	C4—C5—C6—C7	0.3 (2)
C2—N2—C3—C1	−3.79 (15)	C5—C6—C7—F1	179.19 (12)
N1—C1—C3—O2	−176.71 (13)	C5—C6—C7—C8	−0.1 (2)
C4—C1—C3—O2	65.37 (17)	F1—C7—C8—C9	−178.99 (12)
C10—C1—C3—O2	−58.79 (17)	C6—C7—C8—C9	0.3 (2)
N1—C1—C3—N2	3.18 (13)	C7—C8—C9—C4	−0.7 (2)
C4—C1—C3—N2	−114.74 (12)	C5—C4—C9—C8	0.9 (2)
C10—C1—C3—N2	121.09 (12)	C1—C4—C9—C8	−179.95 (12)
N1—C1—C4—C5	−0.87 (17)		

### Hydrogen-bond geometry (Å, °)

**Table:**

*D*—H···*A*	*D*—H	H···*A*	*D*···*A*	*D*—H···*A*
N1—H1···O1^i^	0.88 (2)	2.04 (2)	2.8834 (17)	160.5 (18)
N2—H2···O1^ii^	0.89 (2)	1.96 (2)	2.8318 (17)	165.9 (17)

Symmetry codes: (i) *x*−1/2, −*y*+1/2, −*z*; (ii) *x*+1/2, −*y*+1/2, −*z*.
